# Discovery of novel oestrogen receptor α agonists and antagonists by screening a revisited privileged structure moiety for nuclear receptors

**DOI:** 10.1038/s41598-019-46272-y

**Published:** 2019-07-09

**Authors:** Takahiro Masuya, Masaki Iwamoto, Xiaohui Liu, Ayami Matsushima

**Affiliations:** 1 Laboratory of Structure-Function Biochemistry, Department of Chemistry, Faculty of Science, Kyushu University, Fukuoka, 819-0395 Japan; 2 Laboratory of Molecular and Cellular Biochemistry, Department of Chemistry, Faculty of Science, Kyushu University, Fukuoka, 819-0395 Japan

**Keywords:** Endocrine reproductive disorders, Computational chemistry, Steroid hormones

## Abstract

Bisphenol A (BPA) is used as an industrial raw material for polycarbonate plastics and epoxy resins; however, various concerns have been reported regarding its status as an endocrine-disrupting chemical. BPA interacts not only with oestrogen receptors (ERs) but constitutive androstane receptor, pregnane X receptor, and oestrogen-related receptor γ (ERRγ); therefore, the bisphenol structure represents a privileged structure for the nuclear-receptor superfamily. Here, we screen 127 BPA-related compounds by competitive-binding assay using [^3^H]oestradiol and find that 20 compounds bind to ERα with high affinity. We confirm most of these as ERα agonists; however, four compounds, including bisphenol M and bisphenol P act as novel antagonists. These structures harbour three benzene rings in tandem with terminal hydroxy groups at *para*-positions, with this tandem tri-ring bisphenol structure representing a novel privileged structure for an ERα antagonist. Additionally, we perform an *ab initio* calculation and develop a new clipping method for halogen bonding or non-covalent interaction using DV-Xα evaluation for biomolecules.

## Introduction

Bisphenol A [BPA; HO-C_6_H_4_-C(CH_3_CH_3_)-C_6_H_5_-OH] is a chemical used in the production of polycarbonate plastics and epoxy resins; however, maternal exposure to BPA is considered a developmental and behavioural risk later in life in humans and model animals. BPA binds to oestrogen receptors (ERs) with >1,000-fold weaker affinity than 17β-oestradiol (E2), a natural female hormone, according to traditional radioligand receptor-binding assays^1^. Nevertheless, the biological adverse effects of BPA on experimental animals have been frequently reported^2^, and BPA is recognized as an endocrine-disrupting chemical (EDC). Exposure to “low-dose” BPA (i.e., BPA at much lower doses than those used for typical toxicologic risk assessment) induces reactions in laboratory animals and cultured cells. For example, *in utero* exposure to 2 μg/kg/day or 20 μg/kg/day BPA increases prostate size and weight in adult male mouse offspring^3,4^, with exposure to 20 μg/kg/day BPA also decreasing the efficiency of sperm production by 20%^5^. Furthermore, exposure of 50 pg/mL BPA to isolated and cultured prostates leads to branching and growth similar to that observed following testosterone exposure^4^. Moreover, low-dose BPA affects not only the reproductive systems, but also neurochemistry and structure of the brain, leading to behavioural changes, such as increased aggression, hyperactivity, and learning deficits^6,7^. Such low-dose effects are assumed to be associated with receptor-mediated responses^8^; however, the precise molecular mechanisms remain unknown. The Consortium Linking Academic and Regulatory Insights on BPA Toxicology (CLARITY-BPA) was established by the United States Food and Drug Administration (FDA), National Institute of Environmental Health Sciences (NIEHS), and the National Toxicology Program, and will declare a report integrating findings from the core study and grantee studies in fall 2019^9,10^.

EDCs are assumed to bind nuclear receptors and exert their harmful effects to humans and animals. There are 48 types of nuclear receptors in humans, with all of these representing potential targets of environmental disruptors^11,12^. BPA activates not only ERs but also other nuclear receptors, including constitutive androstane receptor and pregnane X receptor^13–15^, which were originally recognized as xenobiotic receptors and control the innate defence against the exogenous small molecules. These receptors are activated by a variety of chemicals known to induce cytochrome P450 families and bind to the promoter regions of xenobiotic-response elements of the cytochrome P450 family genes^16–18^. BPA binds to oestrogen-related receptor γ (ERRγ) with the highest affinity among the 48 nuclear receptors^19,20^. The crystal structure of the BPA–ERRγ complex is the first of an EDC-bound nuclear receptor and shows multiple intermolecular interactions via benzene rings that promote BPA binding in the ligand-binding pocket of ERRγ^21^. These findings imply that bisphenol and/or benzylphenol structures represent privileged structures for the nuclear-receptor superfamily, with this concept first introduced in 1988 and still recognized as a useful definition for drug-target leads in the field of medicinal chemistry^22,23^.

Various BPA derivatives and their related compounds have been developed, and their use as industrial raw materials has increased substantially. Tetrabromobisphenol and its derivatives are used as flame-retardant materials^24^, and the environmental and health risks of some bisphenol-related chemicals, including bisphenol S and F, have been recently recognized^25–27^; however, many of these have not been examined in detail. Because benzyl-containing structures represent the privileged structure for intrinsic receptors^28,29^, BPA derivatives and/or their related compounds are capable of binding ERs with even higher affinity than BPA. In this study, we screened 127 bisphenol derivatives and their related compounds showing binding affinity for ERα according to receptor-binding assays using radiolabelled [^3^H]E2. Additionally, we screened the transcriptional activity of these compounds in HeLa cells, and found that halogen-containing bisphenol-related compounds exhibited strong ERα-binding affinities. Moreover, we evaluated the structural importance of ligand-receptor interactions between ERα and halogen-containing BPA derivatives using the first principal molecular orbital calculation (DV-Xα cluster method)^30^, combined with our newly developed clipping strategy for **h**alogen bonding^31^ or non-covalent **i**nteraction by D**V**-Xα **e**valuations (i.e., HIVE clip). The aim of this study is to confirm that bisphenol and/or benzylphenol structures are privileged structures for ERα binding and demonstrate the efficacy of our method for assessing utility of halogen bonds to promote ligand binding.

## Results

### ERα preferentially binds bisphenol structures

**Figure 1:**
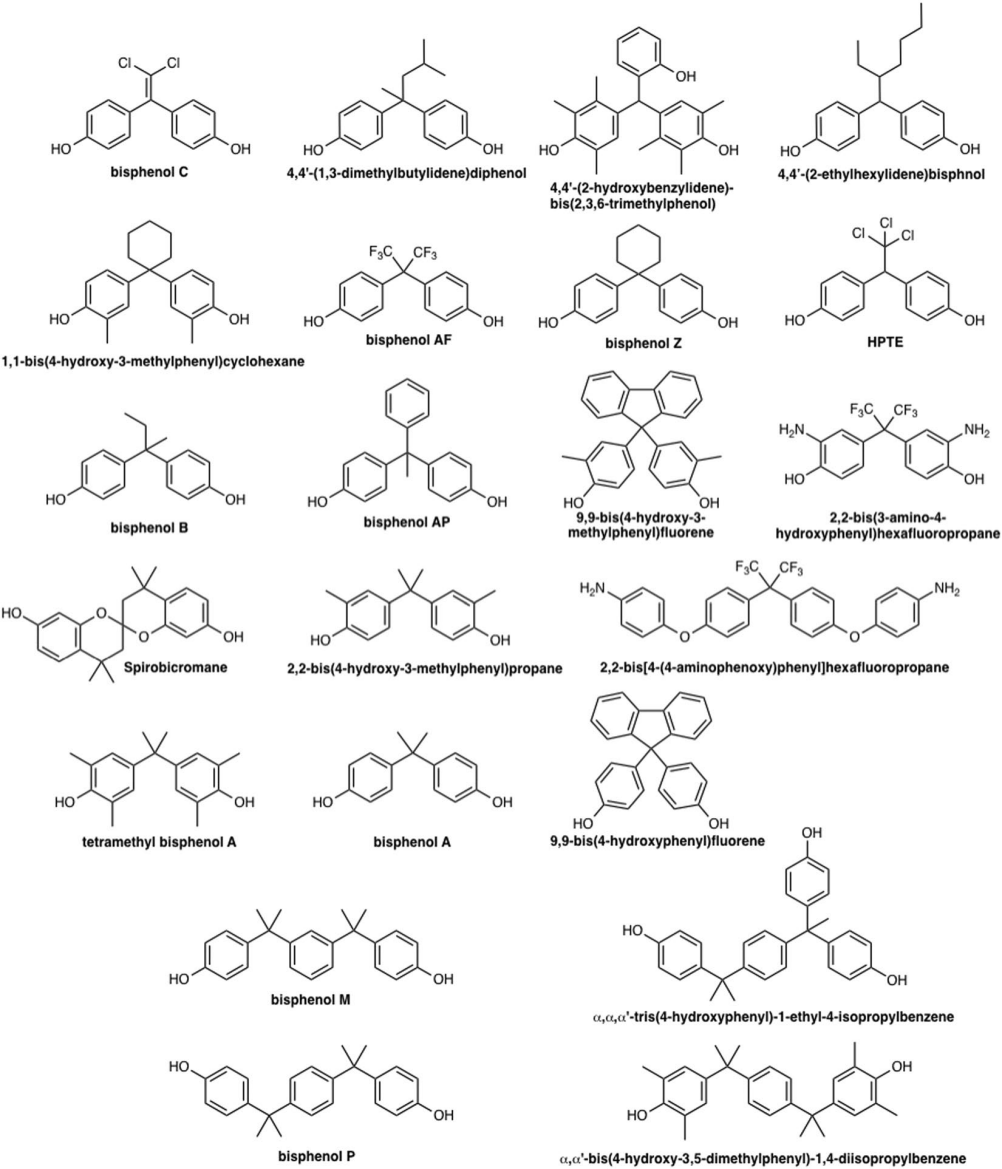
Chemical structures of BPA and its related chemicals. BPA and 20 chemicals exhibiting binding ability stronger than or comparable to BPA are shown, in with BPC having the highest affinity to ERα. 9,9-Bis(4-hydroxyphenyl)fluorine exerted comparable binding ability as that observed for BPA. Four chemicals (bottom) represent tandem tri-ring bisphenols showing ERα–antagonistic activity.

We performed the competitive binding assays using [^3^H]E2 against ERα to evaluate 127 commercially available bisphenol or benzylphenol derivatives, some of which are used as industrial raw materials for polycarbonate plastics. The CAS registry numbers, their common names, and the IUPAC names of all tested chemicals are provided in Supplementary Table 1. Those chemicals include 56 bisphenol-structure-containing chemicals, 10 benzylphenol-structure-containing chemicals, and 61 bisphenol or benzylphenol derivatives with hydroxyl groups substituted primarily with ester groups. Eighteen of the chemicals possess more than three phenyl rings. We found that 70 compounds (>55% of the compounds tested) bound to the ligand-binding domain (LBD) of ERα responsible for ligand-dependent activation function (i.e., the activation function-2 motif). Bisphenol C (BPC) bound to ERα with the highest affinity of all tested compounds, exhibiting a 50% inhibitory concentration (IC_50_) of 2.81 nM, followed by 4,4′-(1,3-dimethylbuthylidene)bisphenol exhibiting an IC_50_ of 5.75 nM. Notably, 20 compounds exhibited stronger or almost the same affinity than BPA as a weak ERα agonist, with their chemical structures shown in Fig. 1 and their respective binding affinity were determined by independently performed competitive binding assays using [^3^H]E2 and summarized in Table 1. Their representative curves and the R^2^ values are shown in Supplementary Fig. 1. The chemical names of the compounds that exhibited lower affinity than BPA are listed in Supplementary Table 2. Sixteen of the 21 compounds (76%; the 20 compounds plus BPA) harboured bisphenol moieties, with a total of 43 of 56 (77%) bisphenol-structure-containing compounds binding to ERα.

**Table 1:** The receptor binding affinity (mean ± SE) of BPA derivatives for estrogen receptor by using [^3^H]17β-oestradiol as a radioligand.

No.	Chemicals	Binding affinity (IC_50_, nM)
E2	estradiol	0.88 ± 0.13
1	bisphenol C	2.81 ± 0.61
2	4,4′-(1,3-dimethylbuthylidene)bisphenol	5.75 ± 1.92
3	4,4′-(2-hydroxybenzylidene)-bis(2,3,6-trimethylphenol)	12.31 ± 7.25
4	4,4-(2-ethylhexylidene)bisphenol	18.46 ± 6.86
5	1,1-bis(4-hydroxy-3-methylphenyl)cyclohexane	38.58 ± 7.16
6	bisphenol AF	53.4 ± 7.3
7	bisphenol M	56.8 ± 11.7
8	bisphenol Z	56.9 ± 0.6
9	2,2-bis(*p*-hydroxyphenyl)-1,1,1- trichloroethane	59.1 ± 1.5
10	α,α,α′-tris(4-hydroxyphenyl)-1-ethyl-4-isopropylbenzene	61.7 ± 10.4
11	bisphenol P	176 ± 35
12	bisphenol B	195 ± 44
13	bisphenol AP	259 ± 41
14	9,9-bis(4-hydroxy-3-methylphenyl)fluorine	321 ± 103
15	2,2-bis(3-amino-4-hydroxyphenyl)hexafluoropropane	334 ± 112
16	spirobicromane	366 ± 20
17	2,2-bis(4-hydroxy-3-methylphenyl)propane	368 ± 22
18	α,α′-bis(4-hydroxy-3,5-dimethylphenyl)-1,4-diisopropylbenzene	733 ± 628
19	2,2-bis[4-(4-aminophenoxy)phenyl]hexafluoropropane	1030 ± 375
20	tetramethyl bisphenol A	1630 ± 300
21	bisphenol A	1780 ± 764
22	9,9-bis(4-hydroxyphenyl)fluorine	2230 ± 202

### Bisphenol-structure-containing chemicals elicit stronger ERα-binding affinity than BPA

**Figure 2:**
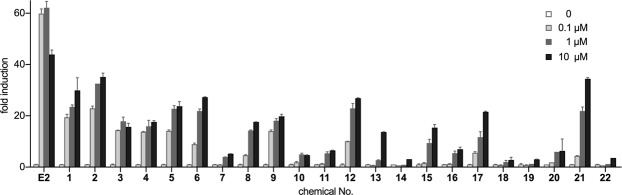
Agonist activities of E2 and BPA-related compounds according to luciferase-reporter assay. White bars indicate the transcriptional activity in the absence of the compounds. Transcriptional activities were evaluated by calculating fold induction from the luminescence versus that observed in the absence of each compound. The dose-dependent transcriptional activities associated with each compound are indicated by bars in light grey (0.1 μM), grey (1 μM), and black (10 μM), respectively. Compound numbers are indicated in Table 1 and Fig. 1.

We used reporter-gene assays to evaluate ERα-related transcriptional activity induced by the 21 compounds. Thirteen of the 21 compounds exhibited agonistic activity, with between 25% and 60% induced activity relative to that by the natural ligand (E2) (Fig. 2). BPC and 4,4′-(1,3-dimethylbuthylidene)bisphenol, as the highest-affinity compounds for ERα (IC_50_ values of 2.81 nM and 5.75 nM, respectively), elicited the highest transcriptional activity (~60%) relative to that of E2, and 13 of the 21 chemicals elicited apparent agonistic activity [BPC, 4,4′-(1,3-dimethylbutylidene)bisphenol, 4,4′-(2-hydroxybenzylidene)-bis(2,3,6-trimethylphenol), 4,4-(2-ethylhexylidene)bisphenol, 1,1-bis(4-hydroxy-3-methylphenyl)cyclohexane, bisphenol AF (BPAF), bisphenol Z, 2,2-bis(*p*-Hydroxyphenyl)-1,1,1- trichloroethane, bisphenol B, bisphenol AP, 2,2-bis(3-amino-4-hydroxyphenyl)hexafluoropropane, 2,2-bis(4-hydroxy-3-methylphenyl)propane, and BPA]. These compounds share the common structural feature of two phenol rings connected by a carbon atom, which is similar to BPA with two methyl groups at the sp3 carbon atom that connects two phenol groups, and 4,4′-(1,3-dimethylbuthylidene)bisphenol, which has a methyl group and an *i*-butyl group at the carbon atom, whereas BPC includes an ethylene structure containing two chlorine atoms. The remaining eight compounds [bisphenol M (BPM), α,α,α′-tris(4-hydroxyphenyl)-1-ethyl-4-isopropylbenzene, bisphenol P (BPP), 9,9-bis(4-hydroxy-3-methylphenyl)fluorine, spirobicromane, α,α′-bis(4-hydroxy-3,5-dimethylphenyl)-1,4-diisopropylbenzene, 2,2-bis[4-(4-aminophenoxy)phenyl]hexafluoropropane, and 9,9-bis(4-hydroxyphenyl)fluorine] elicited weak transcriptional activity.

### Bisphenol derivatives containing three tandem benzene rings exhibit ERα-antagonist activity

**Figure 3:**
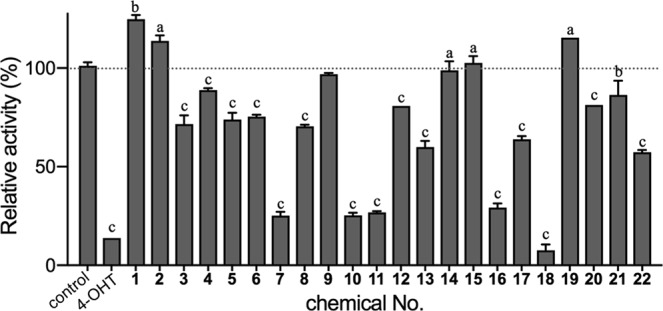
Antagonist activity of 4-OHT and BPA-related compounds according to luciferase-reporter assay. Antagonistic activities by the compounds are indicated as transcriptional activity relative to the luciferase activity induced by 10 nM E2 in the absence of a compound (indicated as control). One-way analysis of variance was performed to analyse significant inhibition of E2-induced activity relative to the activity observed in the absence of the compounds (control). *p < 0.001; **p < 0.0001.

BPM, α,α,α′-tris(4-hydroxyphenyl)-1-ethyl-4-isopropylbenzene, and BPP exhibited strong binding affinity for ERα, with the IC_50_ values of 56.8 nM, 61.7 nM, and 176 nM, respectively; however, these compounds elicited almost no ERα-specific transcriptional activity, suggesting that their binding disrupts the active conformation of ERα, where the C-terminal helix (helix 12) of the LBD is positioned to recruit the coactivators. Therefore, we analysed whether the 21 compounds showing ERα binding affinity inhibit ERα-mediated transcriptional activity induced by a natural ER ligand, E2. We evaluated 10-fold serial dilutions (1 pM to 10 μM) of the compounds in a solution of E2, findings that BPM, α,α,α′-tris(4-hydroxyphenyl)-1-ethyl-4-isopropylbenzene and BPP, spirobicromane, and α,α′-bis(4-hydroxy-3,5-dimethylphenyl)-1,4-diisopropylbenzene inhibited the agonist activity of 1 nM E2 in a dose-dependent manner. At their highest concentration (10 μM), the five compounds reduced E2-mediated ERα activity by up to 90% (Fig. 3). Notably, all of these chemicals, except spirobicromane, share a similar structure involving three benzene rings are tandemly connected by sp^3^ carbon atoms. These structural moieties represent apparent novel privileged structures as ERα antagonists.

### Molecular analysis of the binding mechanism associated with the tandem tri-ring bisphenol structure

**Figure 4:**
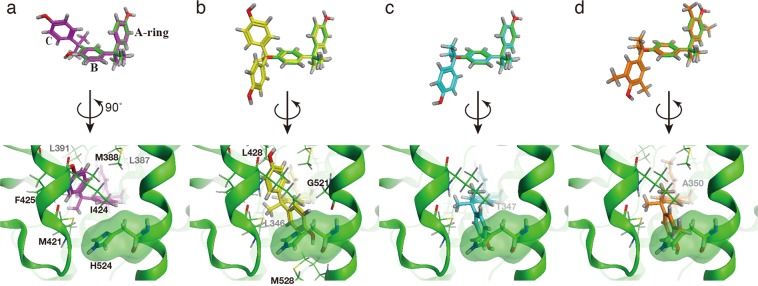
Molecular superposition of tandem tri-ring bisphenols onto the BPA-bound ERα structure. Superposition of tandem tri-ring bisphenols and BPA via the *in silico* Molecular Superpose function show that the tandem tri-ring bisphenols clash with the side chains of the human ERα structure (PDB ID: 3UUA) in its active conformation. BPM (magenta), α,α,α′-tris(4-hydroxyphenyl)-1-ethyl-4-isopropylbenzene (yellow), BPP (blue), and α,α′-bis(4-hydroxy-3,5-dimethylphenyl)-1,4-diisopropylbenzene (orange) are superposed onto the HO-C_6_H_6_-C-C_6_H_6_ moiety of BPA. The molecular surface of the H524 residue located in close proximity to the N-terminal of Helix 11 is illustrated in transparent green. (**a**) BPM clashes with L349, A350 (in Helix 3), L387, M388, L391(in Helix 5), M421, I424, and F425 (in Helix 7), (**b**) α,α,α′-Tris(4-hydroxyphenyl)-1-ethyl-4-isopropylbenzene clashes with L346, A350 (in Helix 3), L387, M388, L391(in Helix 5), M421, I424, F425, L428 (in Helix 7), G521(in Helix 10), H524 and M528 (in Helix 11), (**c**) BPP clashes with L346, T347 (in Helix 3), M421, I424 (in Helix 7), and H524 (in Helix 11), (**d**) α,α′-Bis(4-hydroxy-3,5-dimethylphenyl)-1,4-diisopropylbenzene clashes with A350 (in Helix 3), L387, M388, L391 (in Helix 5), M421, I424 (in Helix 7), and H524 (in Helix 11).

BPM, α,α,α′-tris(4-hydroxyphenyl)-1-ethyl-4-isopropylbenzene, BPP, and α,α′-bis(4-hydroxy-3,5-dimethylphenyl)-1,4-diisopropylbenzene share a common phenylenebis(methylene)bisphenol moiety (i.e., a tandem tri-ring bisphenol structure). We found two crystal structures of bisphenol-bound ERα in the Protein Data Bank (PDB), BPAF-bound ERα in its active conformation (PDB ID: 3UUA) and BPC-bound ERα in its inactive conformation (PDB ID: 3UUC). Molecular superposition of the identified compounds onto the crystal structure of active conformation of ERα was performed to elucidate the key functional moiety associated with their ERα-antagonist activity. The results showed that the terminal phenol rings, (i.e., the C-rings) of the compounds clashed with the H524 side chain located in close proximity to the C-terminal α-helix in the active the conformation of ERα (Fig. 4). Additionally, the A-rings contributing hydrogen bonds between E353 and R394 and the B-rings of the tandem tri-ring bisphenol moieties nearly overlapped those of BPAF. These virtual structures suggested the tandem tri-ring bisphenol moiety as a novel privileged structure for ERα antagonists. In addition, we performed the same analysis using the inactive conformation of ERα with BPC bound. The results indicated that BPM, α,α,α′-tris(4-hydroxyphenyl)-1-ethyl-4-isopropylbenzene, BPP, and α,α′-bis(4-hydroxy-3,5-dimethylphenyl)-1,4-diisopropylbenzene bound to the inactive conformation without clashing with the H524 side chain in the ERα structure (Supplementary Fig. 2). There are precedent crystal structures that showing that the antagonist changes the conformation of ERα receptor by pushing H524 on the C-terminus of Helix 11^32^, therefore these compounds have the potential to induce a similar antagonist conformation.

### Clipping of nitrogen atoms at the *i-1* position is essential for ab initio calculations of LBD conformation

**Figure 5:**
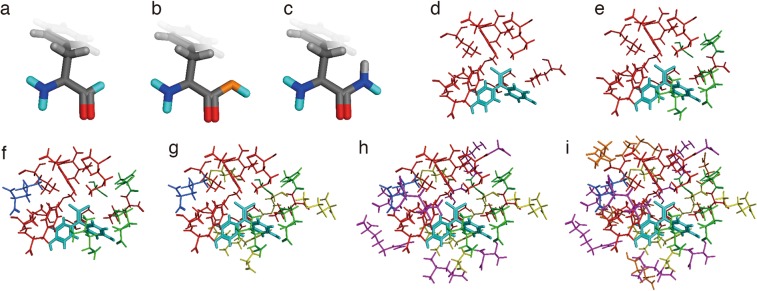
Clipped coordinates and evaluation using three compensating methods or *ab initio* calculation. Terminal atoms were generated by compensating with (**a**) typically utilized protonation methods involving the in-filling of hydrogen atoms (Case 1), (**b**) reconstructing each clipped amino acid residue as an individual amino acid (Case 2), and **c** expanding the selected region to include the nitrogen atom at the *i* − *1* position and protonate conventionally (Case 3), respectively. Clipped atoms from the deposited coordinates of the human ERα crystal structure (PDB ID: 3UUC) were from ranges of (**d**) 4 Å, (**e**) 5 Å, (**f**) 6 Å, (**g**) 7 Å, (**h**) 8 Å, and (**i**) 9 Å.

We used the crystal structure of the BPC-bound ERα LBD (PDB ID: 3UUC) to perform *ab initio* calculations to elucidate the mechanisms associated with the high binding affinity of BPC for the ERα-LBD, which contains two halogen atoms, i.e., two chlorines. We selected amino acid residues near the chlorine atoms from the deposited structure for *ab initio* calculation. To increase the precision of the results, we used three compensating methods for calculations involving the terminal regions. Case 1 involved in-filling hydrogen atoms, Case 2 reconstructed each clipped amino acid residue as individual amino acids, and Case 3 expanded the analysed region to include the nitrogen atom at the *i-1* position along with conventional protonation (Fig. 5a–c).

**Figure 6:**
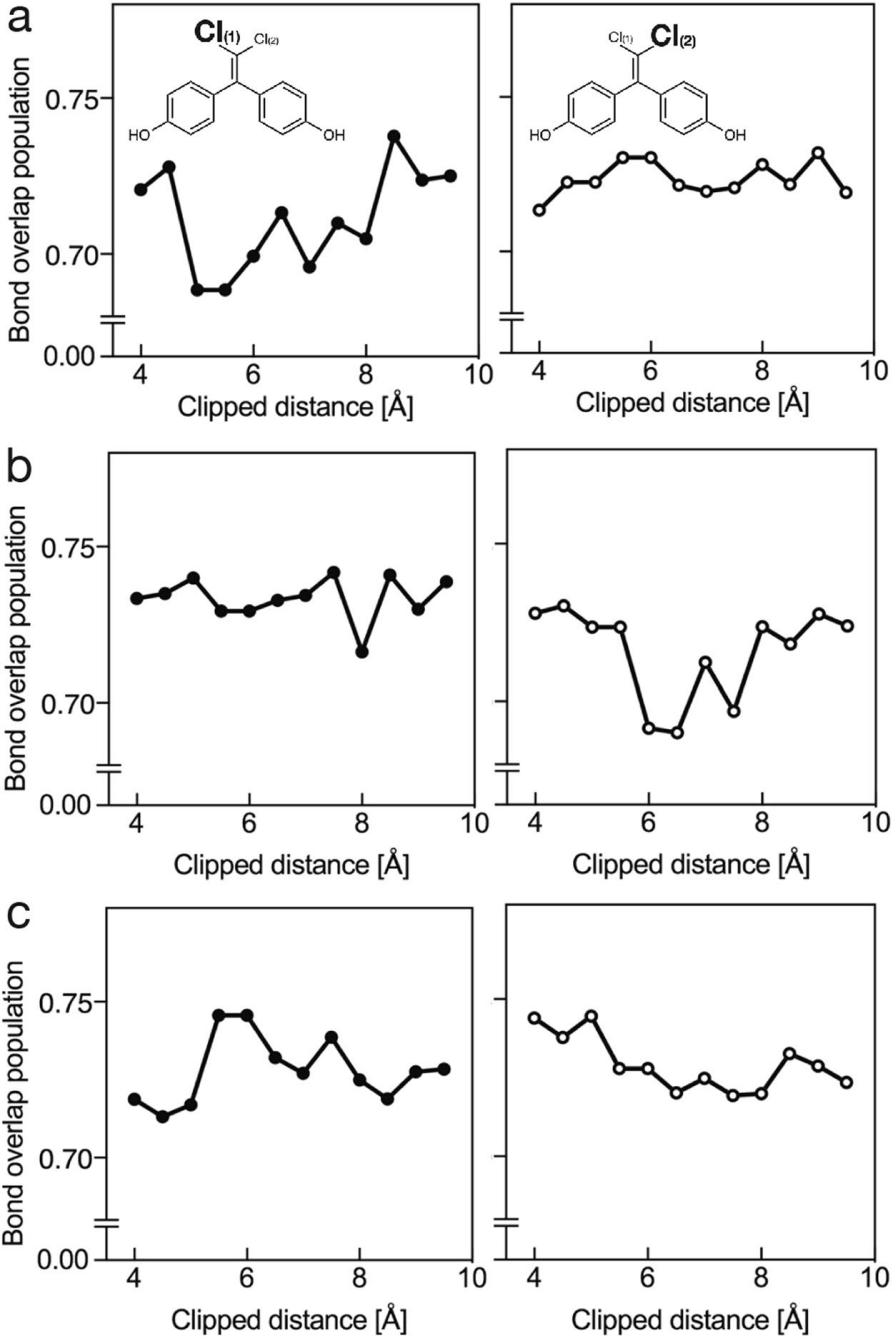
The calculated bond overlap population of chlorine and carbon atoms. The calculated values of bond overlap populations are shown for (**a**) Case 1, (**b**) Case 2, and (**c**) in Case 3 (HIVE chip method). The values of the bond overlap populations converged in Case 3 as upon expansion of the calculated coordinates form the chlorine atoms.

We performed *ab initio* calculations using the coordinate data of from 4 Å to 9 Å for the chlorine atoms of BPC (Fig. 5d–i) in the ERα–BPC complex under the three conditions. The calculated bond overlap population of covalent bonds between chlorines and the carbon atom in the complex are summarized in Fig. 6 and Supplementary Table 3. The average values and their standard deviations of bond overlap population from 7 Å to 8 Å were 0.7072 ± 0.0104, 0.7268 ± 0.0098, and 0.7258 ± 0.0070 in Cases 1, 2, and 3, respectively. The bond overlap population is used to estimate the contribution of covalency in target bonds. The results showed that the compensating method used in Case 3 was useful for *ab initio* calculations of the LBD, because the calculated bond overlap populations were relatively stable and converged upon expansion of the target regions used for calculation. We defined Case 3 as the “**h**alogen or non-covalent **i**nteraction by D**V**-Xα **e**valuation” clipping method (HIVE clip), with this representing a novel method for *ab initio* calculations of regions of large protein structures.

### Determination of ligand-related space constraints for *ab initio* calculations

We evaluated the appropriate distance from the target ligand (BPC) necessary for *ab initio* calculations using the DV-Xα molecular-orbital calculation method. The crystal structure of the ERα-BPC complex was protonated and clipped based on the HIVE method in 0.5-Å increments from 4 Å to 9 Å from the chlorine atoms of BPC. The calculated energy level of the molecular orbitals and the energy diagram density of the chlorine atoms in the clipped region are illustrated in Supplementary Fig. 2. The bond overlap population of the chlorine and carbon atoms converged as we expanded the calculated region. The region from 7 Å to 9 Å, the bond overlap population of C–Cl(1) returned a value of 0.7295, with differences between each bond overlap population and the average value, [|ΔC–Cl(1)| = |C–Cl(1)_n_ − C–Cl(1)_average (7–9 Å)_|]of 0.0020 (at 9 Å) and 0.0164 (at 4.5 Å). All calculated bond overlap populations are summarized in Supplementary Table 4. The results indicated that clipping the coordinates of the residues with protonated nitrogen atoms at the *i–1* position within 7 Å of the chlorine atoms was appropriate for DV-Xα *ab initio* molecular-orbital calculation. Under this condition, the net charges of the chlorine atoms in the ERα-bound structure were −0.18934 and −0.20076, whereas those in the unbound structure were −0.16108 and −0.17064, respectively, suggesting that a net-charge shift occurred upon ERα binding to BPC.

### The lowest unoccupied molecular orbital (LUMO) is located close to bound BPC

**Figure 7:**
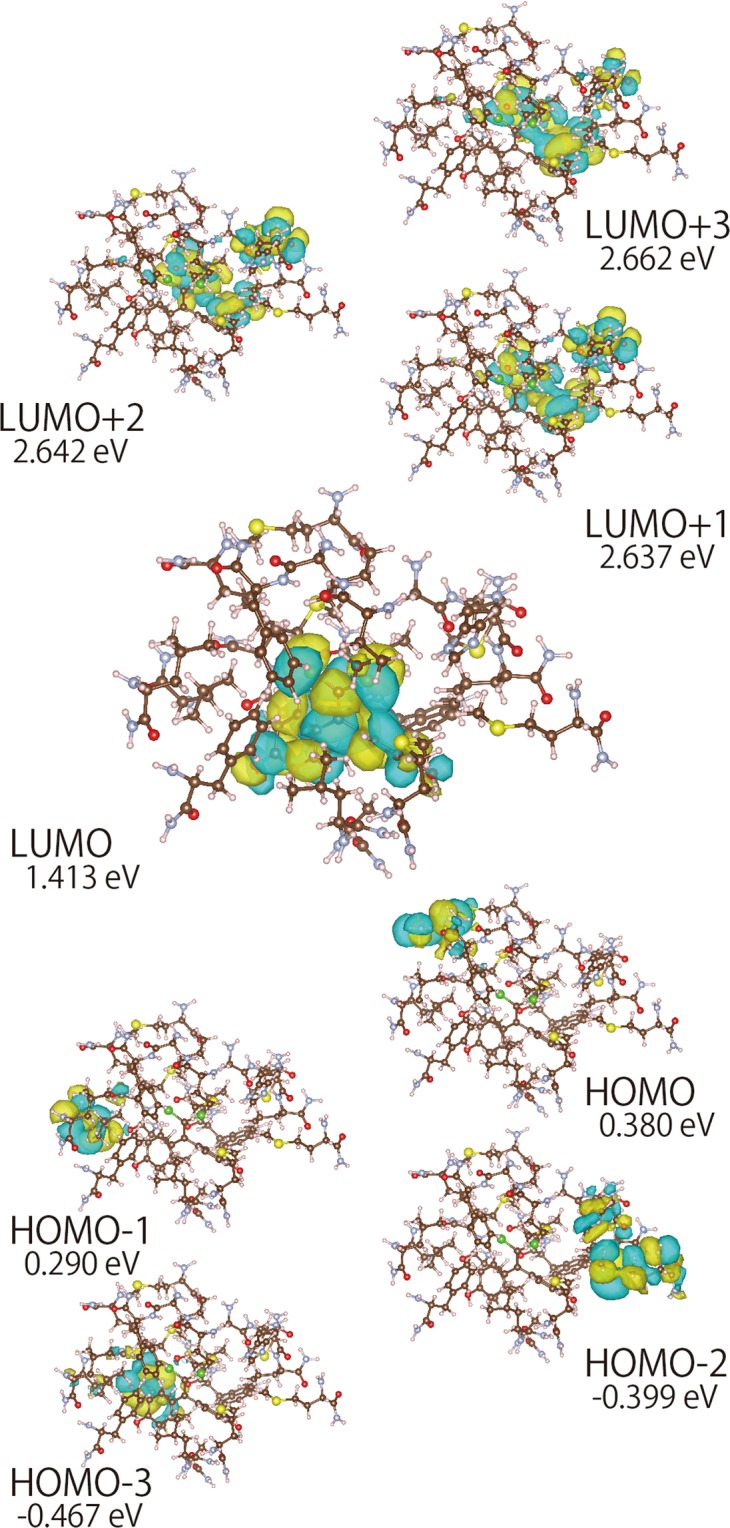
The HOMO and LUMO calculated using the coordinates clipped 7 Å from the chlorine atoms via the HIVE clip method. The calculated molecular orbitals are illustrated as electron clouds. The electron levels of each orbital are indicated under each figure. The LUMO was restricted in close proximity to the ligand in the ERα structure.

We analysed the highest occupied molecular orbital (HOMO) and LUMO from the calculation and illustrated using VESTA^33^ (Fig. 7), revealing a LUMO–HOMO energy gap of 1.0328 eV. Moreover, the LUMO was restricted to a position in close proximity to the BPC ligand in the ERα LBD.

## Discussion

We screened 127 BPA derivatives and their related compounds, revealing that bisphenol derivatives containing three tandem benzene rings (i.e., tandem tri-ring bisphenols) representing novel privileged structures for ERα antagonists. Competitive-binding assays using [^3^H]E2 showed that BPC bound to ER with the highest affinity, followed by 4,4′-(1,3-dimethylbuthylidene)bisphenol, and that BPM, α,α,α′-tris(4-hydroxyphenyl)-1-ethyl-4-isopropylbenzene, BPP, and α,α′-bis(4-hydroxy-3,5-dimethylphenyl)-1,4-diisopropylbenzene also bound to ERα. These tandem tri-ring bisphenols elicited antagonistic effects according to reporter-gene assays in HeLa cells and mainly due to structural conflicts between the C-ring in the tandem tri-ring bisphenols and Helix 11 of ERα. Additionally, bulky groups located on the *sp*^3^-carbon connecting the B-ring and the C-ring displace Helix 12 (i.e., the activation helix) of ERα. HeLa cells are generally used in the first screening according to the Organisation for Economic Co-operation and Development test guidelines No.455 (OECD TG455)^34^; however, oestrogen receptor antagonists frequently show opposing activities in different tissue types, and future studies would provide clues for this unknown mechanism. Moreover, we confirmed that halogen-containing bisphenol derivatives (e.g. BPC) bound to ERα with high affinity. Common methods used for *ab initio* analysis of large biomolecules include fragmentation methods^35,36^, although improved systematic fragmentation methods are required. Our results of the *ab initio* calculation of the ERα–BPC complex indicated that the novel HIVE clip method described in this study was beneficial for evaluating the ligand-bound conformation of the large biomolecules.

Previous studies reported the binding affinities of bisphenol derivatives using competitive binding assay^37–40^, and screening experiments for ERα−related transcriptional activity of ERα were commonly performed using reporter-gene assays. Two studies analysed the transcriptional activities of structurally related BPA derivatives as ERα agonists, with 19 and 55 compounds systematically examined, respectively, although these included neither BPC nor 4,4′-(1,3-dimethylbuthylidene)bisphenol have not been analyzed in both papers^41,42^. The 4-hydroxyl group of the A-phenyl ring and the B-phenyl ring of BPA derivatives are essential for ERα-related transcriptional activity, with the bridging alkyl groups changing their respectively influences^42^. Bisphenol moieties connected by oxime esters show agonistic activity for ERα with suppress cell proliferation in cancer cells^43,44^. The estrogenic activities of contaminants in BPA used for industrial purposes were previously analysed by yeast two-hybrid assays^45^, identifying 4,4′-(1,3-dimethylbuthylidene)bisphenol as exerting 8-fold higher estrogenic activity than laboratory grade BPA. In the present study, our binding assays revealed that 4,4′-(1,3-dimethylbuthylidene)bisphenol bound the to ERα LBD with an IC_50_ value of 5.75 nM, which was sufficient to induce ERα-related activity. A previous study reported high-affinity BPC–ERα binding in ERα-expressing cells, with BPC displaying both agonistic and partially antagonistic activities^46^. The crystal structure of the ERα LBD–BPC complex shows the antagonistic form of BPC binding to ERα^46^. In the present study, we confirmed the high affinity of BPC for the ERα LBD and its role as an ERα agonist to a similar degree as that reported for BPA^46^; however, we did not observe any antagonistic activity by BPC on ERα. A possible explanation might be the use of different cell types, as the previous study used and an HELN cell line stably expressing an ERα-reporter gene^46^, whereas we analysed ERα-related transcriptional activity using transiently transfected HeLa cells. In some cases, high copy numbers introduced by transfected plasmids result in high levels of protein expression, and elevated ERα concentrations in cells might affect their associated transcriptional activity; it is also well known that some oestrogen-receptor ligands act as selective oestrogen receptor modulators (SERMs); therefore further study is needed to clarify this point. We demonstrated for the first time that 9,9-bis(4-hydroxyphenyl)fluorine directly binds to the ERα LBD according to a competitive binding assay using [^3^H]E2. This result agrees with the results of a recent report warning that a BPA substitute, 9,9-bis(4-hydroxyphenyl)fluorine, shows apparent anti-estrogenic activity both *in vitro* and *in vivo*^47^. Furthermore, we found that 9,9-bis(4-hydroxy3-methylphenyl)fluorine bound to the ERα LBD with higher affinity than 9,9-bis(4-hydroxyphenyl)fluorine, suggesting that increased focus should be given to this compound in order to assess to its safety when it is utilized as a BPA alternative.

BPC and BPA share a similar structure; however, BPC binds to the ERα LBD with higher affinity than BPA. Because BPC contains two chlorine atoms, halogen bonds derived from chlorine atoms are possible causes for these strong interactions^31,48,49^. To elucidate the binding mechanism, we performed the *ab initio* molecular orbital calculations using the coordinates of the BPC-bound ERα LBD crystal structure and the HIVE clip method to mimic the peptide bonds. The results showed the calculated LUMO located near the bound ligand moiety, representing the first demonstration of this in a ligand–receptor complex. It is reasonable that LUMO would be restricted near the bound ligand according to use of the HIVE clip method based on its application of a specific clipping strategy using coordinates of protein structures. Further studies including full-electron *ab initio* calculations are needed to reveal the detailed significance of these results.

In summary, we identified a tandem tri-ring bisphenol moiety, such as that found in BPM and BPP, as a novel privileged structure for ERα antagonists. Our results illustrated the LUMO of the BPC–ERα complex crystal structure by *ab initio* calculation according to coordinates selected using our novel HIVE clip method. These results demonstrated the efficacy of this method to accelerate protein-structure analysis and/or simulation for the purpose of screening of ligand–receptor interactions.

## Methods

### Chemicals

17β-E2 (CAS No. 50-28-2; purity, 98.9%) was purchased from Research Biochemicals International (Natick, MA, USA). 4-Hydroxytamoxifen (4-OHT, CAS No. 68047-06-3; purity, 98%) and 2,2-bis(*p*-hydroxyphenyl)-1,1,1-trichloro-ethane (CAS No. 2971-36-0; purity, 98.9%) were obtained from Sigma-Aldrich Inc. (St. Louis, MO, USA). Bisphenol F and hexestrol were obtained from FUJIFILM Wako Pure Chemical Corporation (Osaka, Japan), and remaining 125 chemicals were purchased from Tokyo Chemical Industry Co., Ltd. (Tokyo, Japan). Dimethyl sulfoxide (DMSO) used to dissolve each compound was obtained from Sigma-Aldrich.

### Expression and purification of glutathione S‐transferase (GST)–fused ERα LBD

The ERα cDNA clone was purchased from OriGene Technologies, Inc. (Rockville, MD, USA). For construction of the expression plasmid, the ERα LBD was amplified by polymerase chain reaction and subcloned into the pGEX-6P-1 vector (GE Healthcare, Chicago, IL, USA) to obtain as a GST-fused protein. The GST-fused ERα -LBD was expressed in *Escherichia coli* BL21α cells and purified using Glutathione-Sepharose 4B (GE Healthcare), followed by gel filtration on a Sephadex G-10 column (GE Healthcare).

### Radioligand binding assays

We performed the radioligand binding assays for ERα according to previously reported by method^50^. To validate binding, saturation binding assay was conducted using a tritium-labelled ligand, [^3^H]E2 (4458.5 GBq/mmol; PerkinElmer, Inc., Waltham, MA, USA). GST-ERα-LBD (20 ng) was incubated with a series of [^3^H]E2 (0.1–30 nM) in a total volume of 100 μL of binding buffer [10% glycerol, 10 mM Tris, 1 mM EGTA, 1 mM EDTA, 1 mM sodium vanadate (V), 0.5 mM phenylmethylsulfonyl fluoride, and 0.2 mM leupeptin (pH 7.4)] for 2 h at 20 °C in order to estimate total binding. Specific binding was calculated by subtracting the nonspecific binding determined by addition of 10 μM non-radiolabeled E2 to another set of working solutions from the observed total binding. After incubation, free radioligand was removed by addition and incubation of 100 μL 0.4% dextran-coated charcoal (Sigma-Aldrich) in phosphate-buffered saline (PBS; pH 7.4) on ice for 10 min. After centrifugation for 10 min at 15,000 rpm, the radioactivity of each supernatant was measured using a liquid scintillation counter (LS6500; Beckman Coulter, Fullerton, CA, USA). The data of the calculated specific binding of [^3^H]E2 were assessed by Scatchard plot analysis^51^.

The binding affinities of the test compounds were evaluated by competitive binding assays. Each chemical was dissolved in DMSO to prepare a 1.0-mM stock solution, followed by subsequent incubation of serial dilutions (1 pM to 10 μM) with GST- ERα-LBD (20 ng) and [^3^H]E2 (5 nM, final) for 2 hrs at 20 °C to assess their ability to hinder the binding of [^3^H]E2. Free radioligand was absorbed by 0.4% dextran-coated charcoal for the saturation binding assay and removed by the vacuum filtration system using a 96-well filtration plate (MultiScreenHTS HV; 0.45-mm pore size; Merck KGaA, Darmstadt, Germany). The radioactivities of the eluents were measured using a TopCount NXT system (PerkinElmer) for 3 min/well. To determine the binding affinity of each test compound, IC_50_ values were calculated from the dose-response curves generated by nonlinear regression analysis using the software package Prism (GraphPad Software Inc., La Jolla, CA, USA).

### Luciferase-reporter assay for evaluating agonist activity

HeLa cells were maintained in Eagle’s minimum essential medium (MEM; Nissui Pharmaceutical Co., Ltd, Tokyo, Japan) supplemented with 10% (v/v) foetal bovine serum treated with dextran-coated charcoal at 37 °C under 5% CO_2_. To analyse the agonist activity, HeLa cells were incubated at a density of 5 × 10^5^ cells per 60-mm dish for 24 h, followed by transfection of 3 μg of the reporter plasmid (3 × ERE/pGL4.23) and 1 μg of the ERα expression plasmid (ERα/pcDNA3.1) using Lipofectamine LTX reagent (Thermo Fisher Scientific, Waltham, MA, USA) according to the manufacturer’s instructions. After 24 h, cells were harvested, suspended in Eagle’s MEM, and seeded into 96-well plates at 5 × 10^4^ cells/well, followed by treatment with a series of the test compounds diluted with 1% bovine serum albumin (BSA)/PBS (v/v) to prepare different concentrations (0–10 μM). After a 24-h incubation, luciferase activity was measured using a Luciferase Assay system (Promega, Madison, WI, USA) according to the manufacturer’s instructions. Briefly, cells were lysed using reporter lysis buffer (Promega), and luminescence was measured using a Wallace 1420 ARVOsx multilabel counter (PerkinElmer). Cells treated only with 1% BSA/PBS were used as a vehicle control. Each assay was performed in triplicate and repeated at least three times.

To evaluate antagonistic activity, we examined serial concentrations of test compounds (0–10 μM, final) in the presence of 1 nM or 10 nM concentrations of E2, which normally induces basal levels of ERα-related transcriptional activity^1^.

### Molecular superposition of each antagonist onto ERα-ligand-bound complex structure

The three-dimensional (3D) coordinates of α,α,α′-tris(4-hydroxyphenyl)-1-ethyl-4-isopropylbenzene and α,α′-bis(4-hydroxy-3,5-dimethylphenyl)-1,4-diisopropylbenzene were obtained from CSD-System (ligand IDs: JEHXAW and ACAYIN; CCDC, Cambridge, UK). There were no corresponding entries for of BPM and BPP in CSD-System; therefore, those coordinates were constructed *in silico* using Gaussian 16 (Gaussian, Inc., Wallingford CT, USA) with the basis set of 6–31G. We superposed these structures onto BPAF in the BPAF–ERα complex (PDB ID: 3UUA) and BPC in the BPC–ERα complex (PDB ID: 3UUC), respectively, using the Molecular Superpose function in the Molecular Operating Environment (MOE) platform (Chemical Computing Group, Montreal, Canada).

### First principal molecular orbital calculation

We performed the first principal molecular orbital calculation (i.e., *ab initio* calculation) in order to illuminate the strong affinity of halogen-containing bisphenol derivatives. We used the DV-Xα cluster method^30^, to calculate the electron states of a solo BPC compound and the BPC–ERα structure using Slater’s exchange potential and numerical basis functions. We calculated bond overlap population and net charge using Mulliken population analysis. The bond overlap population obtained using this method represents a measure of the covalent bonding between target atoms. The 3D coordinates of the BPC–ERα complex were obtained from the PDB (PDB ID: 3UUC).

## Supplementary information

Supplementary materials
