# Supplementary material for: Discovery of novel oestrogen receptor α agonists and antagonists by screening a revisited privileged structure moiety for nuclear receptors
Source: Sci Rep. 2019 Jul 9;9:9954. doi: 10.1038/s41598-019-46272-y (PMC6616570; doi:10.1038/s41598-019-46272-y)
Supplement: Supplementary file 1 — Supplementary materials [file 41598_2019_46272_MOESM1_ESM.pdf]

**Supplementary information**

# Discovery of novel oestrogen receptor $\alpha$ agonists and antagonists by screening a revisited privileged structure moiety for nuclear receptors

Takahiro Masuya[1], Masaki Iwamoto[1], Xiaohui Liu[2], and Ayami Matsushima[1]*

[1]Laboratory of Structure-Function Biochemistry, Department of Chemistry, Faculty of Science, Kyushu University, Fukuoka 819-0395, Japan

[2]Laboratory of Molecular and Cellular Biochemistry, Department of Chemistry, Faculty of Science, Kyushu University, Fukuoka 819-0395, Japan

* Correspondence and requests for materials should be addressed to A.M. (E-mail: ayami@chem.kyushu-univ.jp).

*Author to whom correspondence should be addressed; E-Mail: ayami@chem.kyushu-univ.jp; Tel: +81-92-802-4159/Fax: +81-92-802-4126.

# SUPPLEMENTARY TABLES

## Supplementary table 1.

The list of compounds name, CAS Registry No., and IUPAC name of all chemicals analyzed their binding ability to ERa by the competitive binding assay in this study.

| compounds | CAS Registry No. | IUPAC name |
|---|---|---|
| 4,4',4''-trihydroxytriphenylmethane | 603-44-1 | 4,4',4"-methanetriyltriphenol |
| tetrachloro bisphenol A | 79-95-8 | 4,4'-(propane-2,2-diyl)bis(2,6-dichlorophenol) |
| tetrabromo bisphenol A | 79-94-7 | 4,4'-(propane-2,2-diyl)bis(2,6-dibromophenol) |
| bisphenol B | 77-40-7 | 4,4'-(butane-2,2-diyl)diphenol |
| bisphenol AP | 1571-75-1 | 4,4'-(1-phenylethane-1,1-diyl)diphenol |
| tetramethyl bisphenol A | 5613-46-7 | 4,4'-(propane-2,2-diyl)bis(2,6-dimethylphenol) |
| 2,2-bis(4-hydroxy-3-methylphenyl)propane | 79-97-0 | 4,4'-(propane-2,2-diyl)bis(2-methylphenol) |
| 1,1',1''-tris(4-hydroxyphenyl)ethane | 27955-94-8 | 4,4',4"-(ethane-1,1,1-triyl)triphenol |
| 4-α-cumyl phenol | 599-64-4 | 4-(2-phenylpropan-2-yl)phenol |
| bisphenol P | 2167-51-3 | 4,4'-(1,4-phenylenebis(propane-2,2-diyl))diphenol |
| bisphenol C | 14868-03-2 | 4,4'-(2,2-dichloroethene-1,1-diyl)diphenol |
| bisphenol A | 80-05-7 | 4,4'-(propane-2,2-diyl)diphenol |
| hexachlorophene | 70-30-4 | 6,6'-methylenebis(2,4,5-trichlorophenol) |
| α,α,α'-tris(4-hydroxyphenyl)-1-ethyl-4-isopropylbenzene | 110726-28-8 | 4,4'-(1-(4-(2-(4-hydroxyphenyl)propan-2-yl)phenyl)ethane-1,1-diyl)diphenol |
| α,α'-bis(4-aminophenyl)-1,4-diisopropylbenzene | 2716-10-1 | 4,4'-(1,4-phenylenebis(propane-2,2-diyl))dianiline |
| 2,2-bis(3-cyclohexyl-4-hydroxyphenyl)propane | 57100-74-0 | 4,4'-(propane-2,2-diyl)bis(2-cyclohexylphenol) |
| 2,2-bis(2-hydroxy-5-biphenylyl)propane | 24038-68-4 | 5,5"-(propane-2,2-diyl)bis(([1,1'-biphenyl]-2-ol)) |
| 2,2-bis(4-glycidyloxyphenyl)propane | 1675-54-3 | 2,2'-(((propane-2,2-diylbis(4,1-phenylene))bis(oxy))bis(methylene))bis(oxirane) |
| bisphenol A diacetate | 10192-62-8 | propane-2,2-diylbis(4,1-phenylene) diacetate |
| tetrabromobisphenol A bis(2-hydroxyethyl)ether | 4162-45-2 | 2,2'-((propane-2,2-diylbis(2,6-dibromo-4,1-phenylene))bis(oxy))diethanol |
| 2,2-bis(4-hydroxy-3-isopropylphenyl)propane | 127-54-8 | 4,4'-(propane-2,2-diyl)bis(2-isopropylphenol) |
| 4,4'-Isopropylidenediphenoxyacetic acid | 3539-42-2 | 2,2'-((propane-2,2-diylbis(4,1-phenylene))bis(oxy))diacetic acid |
| 2,2-bis[4-(4-aminophenoxy)-phenyl]propane | 13080-86-9 | 4,4'-((propane-2,2-diylbis(4,1-phenylene))bis(oxy))dianiline |
| α,α'-bis(4-hydroxy-3,5-dimethylphenyl)-1,4-diisopropylbenzene | 36395-57-0 | 4,4'-(1,4-phenylenebis(propane-2,2-diyl))bis(2,6-dimethylphenol) |
| 2,2-bis(4-chloroformyloxyphenyl)propane | 2024-88-6 | propane-2,2-diylbis(4,1-phenylene) dicarbonochloridate |
| 2,2-bis(3-sec-butyl-4-hydroxyphenyl)propane | 32113-46-5 | 4,4'-(propane-2,2-diyl)bis(2-(sec-butyl)phenol) |
| bisphenol F | 620-92-8 | 4,4'-methylenediphenol |
| hexestrol | 84-16-2 | 4,4'-(hexane-3,4-diyl)diphenol |
| 2,2-bis(4-cyanatophenyl)propane | 1156-51-0 | 4,4'-(propane-2,2-diyl)bis(cyanatobenzene) |
| isoliquirtigenin | 961-29-5 | (E)-1-(2,4-dihydroxyphenyl)-3-(4-hydroxyphenyl)prop-2-en-1-one |
| 4,4'-methylenebis(2-methylphenol) | 2467-25-6 | 4,4'-methylenebis(2-methylphenol) |
| 2,3,4-trihydroxydiphenylmethane | 17345-66-3 | 4-benzylbenzene-1,2,3-triol |
| bisphenol E | 2081-08-5 | 4,4'-(ethane-1,1-diyl)diphenol |
| phenolphthalein | 81-90-3 | 2-[bis(4-hydroxyphenyl)methyl]benzoic acid |
| HPTE | 2971-36-0 | 4,4'-(2,2,2-trichloroethane-1,1-diyl)diphenol |
| 2,2-bis(3-amino-4-hydroxyphenyl)hexafluoropropane | 83558-87-6 | 4,4'-(perfluoropropane-2,2-diyl)bis(2-aminophenol) |

| | | |
|---|---|---|
| 2,2-bis(3-aminophenyl)hexafluoropropane | 47250-53-3 | 3,3'-(perfluoropropane-2,2-diyl)dianiline |
| 2,2-bis(3-amino-4-methylphenyl)hexafluoropropane | 116325-74-7 | 5,5'-(perfluoropropane-2,2-diyl)bis(2-methylaniline) |
| 2,2-bis(4-aminophenyl)hexafluoropropane | 1095-78-9 | 4,4'-(perfluoropropane-2,2-diyl)dianiline |
| 2,2-bis[4-(4-aminophenoxy)phenyl]hexafluoropropane | 69563-88-8 | 4,4'-(((perfluoropropane-2,2-diyl)bis(4,1-phenylene))bis(oxy))dianiline |
| bisphenol AF | 1478-61-1 | 4,4'-(perfluoropropane-2,2-diyl)diphenol |
| 2,2-bis(4-carboxyphenyl)hexafluoropropane | 1171-47-7 | 4,4'-(perfluoropropane-2,2-diyl)dibenzoic acid |
| hexafluoro-2,2-diphenylpropane | 83558-76-3 | (perfluoropropane-2,2-diyl)dibenzene |
| 1,1-bis(3-cyclohexyl-4-hydroxyphenyl)cyclohexane | 4221-68-5 | 4,4'-(cyclohexane-1,1-diyl)bis(2-cyclohexylphenol) |
| 9,9-bis(4-aminophenyl)fluorene | 15499-84-0 | 4,4'-(9h-fluorene-9,9-diyl)dianiline |
| 4,4'-(2-hydroxybenzylidene)-bis(2,3,6-trimethylphenol) | 184355-68-8 | 4,4'-((2-hydroxyphenyl)methylene)bis(2,3,6-trimethylphenol) |
| 4,4'-(1,3-dimethylbutylidene)diphenol | 6807-17-6 | 4,4'-(4-methylpentane-2,2-diyl)diphenol |
| 9,9-bis(4-hydroxyphenyl)fluorene | 3236-71-3 | 4,4'-(9h-fluorene-9,9-diyl)diphenol |
| 9,9-bis(4-hydroxy-3-methylphenyl)fluorene | 88938-12-9 | 4,4'-(9h-fluorene-9,9-diyl)bis(2-methylphenol) |
| 4,4'-(2-Ethylhexylidene)diphenol | 74462-02-5 | 4,4'-(2-ethylhexane-1,1-diyl)diphenol |
| 9,9-bis[4-(2-hydroxyethoxy)phenyl]fluorene | 117344-32-8 | 2,2'-(((9h-fluorene-9,9-diyl)bis(4,1-phenylene))bis(oxy))diethanol |
| 1,1-bis(4-hydroxy-3-methylphenyl)cyclohexane | 2362-14-3 | 4,4'-(cyclohexane-1,1-diyl)bis(2-methylphenol) |
| 1,1-bis(4-aminophenyl)cyclohexane | 3282-99-3 | 4,4'-(cyclohexane-1,1-diyl)dianiline |
| bisphenol Z | 843-55-0 | 4,4'-(cyclohexane-1,1-diyl)diphenol |
| 1,3-bis[2-(4-hydroxyphenyl)-2-propyl]benzene | 13595-25-0 | 4,4'-(1,3-phenylenebis(propane-2,2-diyl))diphenol |
| diphenylsilanediol | 947-42-2 | diphenylsilanediol |
| 4-(phenylazo)phenol | 20714-70-9 | (E)-4-(phenyldiazenyl)phenol |
| Resveratrol | 501-36-0 | (E)-5-(4-hydroxystyryl)benzene-1,3-diol |
| Spirobicromane | 3127-14-8 | 4,4,4',4'-tetramethyl-2,2'-spirobi[chroman]-7,7'-diol |
| 6,6',7,7'-tetrahydroxy-4,4,4',4'-tetramethyl-2,2'-spirobichroman | 32737-35-2 | 4,4,4',4'-tetramethyl-2,2'-spirobi[chroman]-6,6',7,7'-tetraol |
| 4,4’-dihydroxybenzophenone | 611-99-4 | bis(4-hydroxyphenyl)methanone |
| 4,4'-dimethoxybenzophenone | 90-96-0 | bis(4-methoxyphenyl)methanone |
| 2,3',4,4'-tetrahydroxybenzophenone | 61445-50-9 | (2,4-dihydroxyphenyl)(3,4-dihydroxyphenyl)methanone |
| 2,2',4,4'-tetrahydroxybenzophenone | 131-55-5 | bis(2,4-dihydroxyphenyl)methanone |
| 4,4'-difluorobenzophenone | 345-92-6 | bis(4-fluorophenyl)methanone |
| 2,2'-dihydroxy-4,4'-dimethoxybenzophenone | 131-54-4 | bis(2-hydroxy-4-methoxyphenyl)methanone |
| 4,4'-dichlorobenzophenone | 90-98-2 | bis(4-chlorophenyl)methanone |
| benzophenone-2,4'-dicarboxylic acid monohydrate | 85-58-5 | 2-(4-carboxybenzoyl)benzoic acid |
| 2,4'-difluorobenzophenone | 342-25-6 | (2-fluorophenyl)(4-fluorophenyl)methanone |
| 4,4'-diaminobenzophenone | 611-98-3 | bis(4-aminophenyl)methanone |
| 4-benzoyl 4'-methyldiphenyl sulfide | 83846-85-9 | phenyl(4-(p-tolylthio)phenyl)methanone |
| benzophenone-4,4'-dicarboxylic acid | 964-68-1 | 4,4'-carbonyldibenzoic acid |
| 2,2'-dihydroxy-4-methoxybenzophenone | 131-53-3 | (2-hydroxy-4-methoxyphenyl)(2-hydroxyphenyl)methanone |
| 2,4'-dichlorobenzophenone | 85-29-0 | (2-chlorophenyl)(4-chlorophenyl)methanone |
| 2,4,4'-trihydroxybenzophenone | 1470-79-7 | (2,4-dihydroxyphenyl)(4-hydroxyphenyl)methanone |
| 2,2'-dihydroxybenzophenone | 835-11-0 | bis(2-hydroxyphenyl)methanone |
| 3,3'-dinitrobenzophenone | 21222-05-9 | bis(3-nitrophenyl)methanone |
| 3,3'-diaminobenzophenone | 611-79-0 | bis(3-aminophenyl)methanone |
| 2-Amino-2',5-dichlorobenzophenone | 2958-36-3 | (2-amino-5-chlorophenyl)(2-chlorophenyl)methanone |
| 2,2',4-trimethoxybenzophenone | 33077-87-1 | (2,4-dimethoxyphenyl)(2-methoxyphenyl)methanone |
| 4,4'-bis(methylamino)benzophenone | 3708-39-2 | bis(4-(methylamino)phenyl)methanone |
| benzophenone | 119-61-9 | benzophenone |

| | | |
|---|---|---|
| 2,3,4,4'-tetrahydroxybenzophenone | 31127-54-5 | (4-hydroxyphenyl)(2,3,4-trihydroxyphenyl)methanone |
| 4,4'-methylenebis(2,6-di-tert-butylphenol) | 118-82-1 | 4,4'-methylenebis(2,6-di-tert-butylphenol) |
| Methylenedisalicylic acid | 122-25-8 | 5,5'-methylenebis(2-hydroxybenzoic acid) |
| bis(3-ethyl-5-methyl-4-maleimidophenyl)methane | 105391-33-1 | 1,1'-(methylenebis(2-ethyl-6-methyl-4,1-phenylene))bis(1h-pyrrole-2,5-dione) |
| 2,2'-methylenebis(4-chlorophenol) | 97-23-4 | 2,2'-methylenebis(4-chlorophenol) |
| 4,4'-bismaleimidodiphenylmethane | 13676-54-5 | 1,1'-(methylenebis(4,1-phenylene))bis(1h-pyrrole-2,5-dione) |
| 4,4'-methylenebis(2-ethyl-6-methylaniline) | 19900-72-2 | 4,4'-methylenebis(2-ethyl-6-methylaniline) |
| 4,4'-diamino-3,3'-dimethyldiphenylmethane | 838-88-0 | 4,4'-methylenebis(2-methylaniline) |
| 4,4'-diaminodiphenylmethane | 101-77-9 | 4,4'-methylenedianiline |
| bis[4-dimethylamino)-phenyl]methane | 101-61-1 | 4,4'-methylenebis(N,N-dimethylaniline) |
| 2,2'-methylenebis(6-tert-butyl-4-ethylphenol) | 88-24-4 | 6,6'-methylenebis(2-(tert-butyl)-4-ethylphenol) |
| 4,4'-methylenebis(2-chloroaniline) | 101-14-4 | 4,4'-methylenebis(2-chloroaniline) |
| 4,4'-methylenebis(2,6-dimethylphenol) | 5384-21-4 | 4,4'-methylenebis(2,6-dimethylphenol) |
| 3,4'-diaminodiphenylmethane | 19430-83-2 | 3-(4-aminobenzyl)aniline |
| 2,2'-methylenebis(6-tert-butyl-p-cresol) | 119-47-1 | 6,6'-methylenebis(2-(tert-butyl)-4-methylphenol) |
| 4,4'-dinitrodiphenylmethane | 1817-74-9 | bis(4-nitrophenyl)methane |
| bis(4-amino-2,3-dichlorophenyl)methane | 42240-73-3 | 4,4'-methylenebis(2,3-dichloroaniline) |
| 2,2'-methylenebis(4-methylphenol) | 3236-63-3 | 2,2'-methylenebis(4-methylphenol) |
| 2,4'-dihydroxydiphenylmethane | 2467-03-0 | 2-(4-hydroxybenzyl)phenol |
| 4,4'-difluorodiphenylmethane | 457-68-1 | bis(4-fluorophenyl)methane |
| 4,4'-diphenylmethane diisocyanate, (4,4'-methylenebis(phenyl Isocyanate)) | 101-68-8 | bis(4-isocyanatophenyl)methane |
| 4,4'-diisocyanato-3,3'-dimethyldiphenylmethane | 139-25-3 | bis(4-isocyanato-3-methylphenyl)methane |
| 3,3'-diaminodiphenylmethane | 19471-12-6 | 3,3'-methylenedianiline |
| 2,2'-dihydroxydiphenylmethane | 2467-02-9 | 2,2'-methylenediphenol |
| 4,4'-dihydroxytetraphenylmethane | 1844-01-5 | 4,4'-(diphenylmethylene)diphenol |
| 2,3,4,4'-tetrahydroxydiphenylmethane | 174462-43-2 | 4-(4-hydroxybenzyl)benzene-1,2,3-triol |
| 3,3'-dihydroxydiphenylamine | 65461-91-8 | 3,3'-azanediyldiphenol |
| 2,2'-dihydroxydiphenyl ether | 15764-52-0 | 2,2'-oxydiphenol |
| bis(4-hydroxyphenyl) sulfide | 2664-63-3 | 4,4'-thiodiphenol |
| 4,4'-dihydroxydiphenyl ether | 1965-09-9 | 4,4'-oxydiphenol |
| 4-phenylphenol | 92-69-3 | [1,1'-biphenyl]-4-ol |
| bis(4-hydroxyphenyl) sulfone (bpS) | 80-09-1 | 4,4'-sulfonyldiphenol |
| 4,4'-biphenol | 92-88-6 | [1,1'-biphenyl]-4,4'-diol |
| 4-methoxybiphenyl | 613-37-6 | 4-methoxy-1,1'-biphenyl |
| biphenyl | 92-52-4 | 1,1'-biphenyl |
| 4,4'-diethoxybiphenyl | 7168-54-9 | 4,4'-diethoxy-1,1'-biphenyl |
| 4-hydroxy-4'-methoxybiphenyl | 16881-71-3 | 4'-methoxy-[1,1'-biphenyl]-4-ol |
| 4-benzylphenol | 101-53-1 | 4-benzylphenol |
| 4-triphenylmethylphenol | 978-86-9 | 4-tritylphenol |
| p-naphtholbenzein | 145-50-6 | (E)-4-((4-hydroxynaphthalen-1-yl)(phenyl)methylene)naphthalen-1(4h)-one |
| 3,3',5-triiodo-L-thyronine | 6893-02-3 | (S)-2-amino-3-(4-(4-hydroxy-3-iodophenoxy)-3,5-diiodophenyl)propanoic acid |
| p,p-DDE | 72-55-9 | 1,1-dichloro-2,2-bis(4-chlorophenyl)ethene |
| 4-hydroxybenzoic acid phenyl ester | 17696-62-7 | phenyl 4-hydroxybenzoate |
| 4-hydroxybenzoic acid benzyl ester | 94-18-8 | benzyl 4-hydroxybenzoate |

**Supplementary table 2**. The list of compounds exhibiting weaker binding ability than BPA against ERα in addition to table 1 ($IC_{50}$: > 1780 nM) by the competitive binding assay using [$^3$H] 17β-estradiol as a radioligand.

| compounds | compounds |
|---|---|
| tetrachloro bisphenol A | 2,2-bis(4-aminophenyl)hexafluoropropane |
| tetrabromo bisphenol A | hexafluoro-2,2-diphenylpropane |
| tetramethyl bisphenol A | 1,1-bis(3-cyclohexyl-4-hydroxyphenyl)cyclohexane |
| 1,1’,1”-Tris(4-hydroxyphenyl)ethane | 9,9-bis(4-aminophenyl)fluorene |
| 4-α-cumyl phenol | 9,9-bis[4-(2-hydroxyethoxy)phenyl]fluorene |
| hexachlorophene | 1,1-bis(4-aminophenyl)cyclohexane |
| α,α'-bis(4-aminophenyl)-1,4-diisopropylbenzene | 4-(phenylazo)phenol |
| 2,2-bis(3-cyclohexyl-4-hydroxyphenyl)propane | 4,4’-dihydroxybenzophenone |
| 2,2-bis(2-hydroxy-5-biphenylyl)propane | 2,3',4,4'-tetrahydroxybenzophenone |
| bisphenol A diacetate | 2,4,4'-trihydroxybenzophenone |
| tetrabromobisphenol A bis(2-hydroxyethyl)ether | 2,2',4,4'-tetrahydroxybenzophenone |
| 2,2-bis(4-hydroxy-3-isopropylphenyl)propane | 2,2'-methylenebis(4-chlorophenol) |
| 2,2-bis[4-(4-aminophenoxy)-phenyl]propane | 4,4'-diamino-3,3'-dimethyldiphenylmethane |
| 2,2-bis(4-chloroformyloxyphenyl)propane | 4,4'-dihydroxytetraphenylmethane |
| 2,2-bis(3-sec-butyl-4-hydroxyphenyl)propane | bis(4-hydroxyphenyl) sulfide |
| bisphenol F | 4,4'-dihydroxydiphenyl ether |
| 2,2-bis(4-cyanatophenyl)propane | 4-phenylphenol |
| isoliquirtigenin | 4,4'-biphenol |
| 4,4'-methylenebis(2-methylphenol) | 4-hydroxy-4'-methoxybiphenyl |
| bisphenol E | 4-benzylphenol |
| phenolphthalin | 4-triphenylmethylphenol |
| 2,2-bis(3-aminophenyl)hexafluoropropane | 4-hydroxybenzoic acid phenyl ester |
| 2,2-bis(3-amino-4-methylphenyl)hexafluoropropane | 4-hydroxybenzoic acid benzyl ester |

**Supplementary table 3**.

The values of each bond overlap population of covalent bonds between Cl and C in ERα/BPC complex by *ab initio* calculation.

| Distance | Bond overlap populations under the condition of | | | | | |
|---|---|---|---|---|---|---|
| | Case 1 | | Case 2 | | Case 3 | |
| (Å) | $Cl_{(1)}$ | $Cl_{(2)}$ | $Cl_{(1)}$ | $Cl_{(2)}$ | $Cl_{(1)}$ | $Cl_{(2)}$ |
| 4.0 | 0.7206 | 0.7279 | 0.7334 | 0.7134 | 0.7187 | 0.7440 |
| 4.5 | 0.7279 | 0.7303 | 0.7350 | 0.7224 | 0.7131 | 0.7378 |
| 5.0 | 0.6885 | 0.7235 | 0.7399 | 0.7225 | 0.7170 | 0.7446 |
| 5.5 | 0.6885 | 0.7235 | 0.7293 | 0.7304 | 0.7456 | 0.7279 |
| 6.0 | 0.6994 | 0.6915 | 0.7293 | 0.7304 | 0.7456 | 0.7279 |
| 6.5 | 0.7133 | 0.6901 | 0.7328 | 0.7215 | 0.7321 | 0.7202 |
| 7.0 | 0.6959 | 0.7123 | 0.7344 | 0.7195 | 0.7271 | 0.7248 |
| 7.5 | 0.7099 | 0.6968 | 0.7417 | 0.7207 | 0.7386 | 0.7193 |
| 8.0 | 0.7049 | 0.7236 | 0.7162 | 0.7281 | 0.7249 | 0.7200 |
| 8.5 | 0.7378 | 0.7181 | 0.7409 | 0.7217 | 0.7189 | 0.7327 |
| 9.0 | 0.7238 | 0.7277 | 0.7300 | 0.7320 | 0.7275 | 0.7287 |
| 9.5 | 0.7251 | 0.7239 | 0.7388 | 0.7191 | 0.7284 | 0.7235 |

**Supplemental table 4.**

The calculated bond overlap population of each chlorine and adjacent carbon atom; C-Cl(1) and C-Cl(2). [a] | ΔC-Cl(1) | = | $C\text{-}Cl(1)_n - C\text{-}Cl(1)_{average\ (7\text{-}9\ Å)}$ |. [b] | ΔC-Cl(2) | = | $C\text{-}Cl(2)_n - C\text{-}Cl(2)_{average\ (7\text{-}9\ Å)}$ |.

| Clipped distance from chlorine atoms (Å) | C-Cl(1) | \|ΔC-Cl(1)\|[a] | C-Cl(2) | \|ΔC-Cl(2)\|[b] | Number of atoms |
|---|---|---|---|---|---|
| 4 | 0.7187 | - | 0.744 | - | 230 |
| 4.5 | 0.7131 | 0.0164 | 0.7378 | 0.0146 | 251 |
| 4 | 0.7170 | 0.0125 | 0.7446 | 0.0214 | 302 |
| 5.5 | 0.7456 | 0.0161 | 0.7279 | 0.0047 | 325 |
| 6 | 0.7456 | 0.0161 | 0.7279 | 0.0047 | 325 |
| 6.5 | 0.7321 | 0.0026 | 0.7202 | 0.0030 | 365 |
| 7 | 0.7271 | 0.0024 | 0.7248 | 0.0016 | 441 |
| 7.5 | 0.7386 | 0.0091 | 0.7193 | 0.0039 | 503 |
| 8 | 0.7249 | 0.0046 | 0.7200 | 0.0032 | 579 |
| 9 | 0.7275 | 0.0020 | 0.7287 | 0.0055 | 682 |

# SUPPLEMENTARY FIGURES

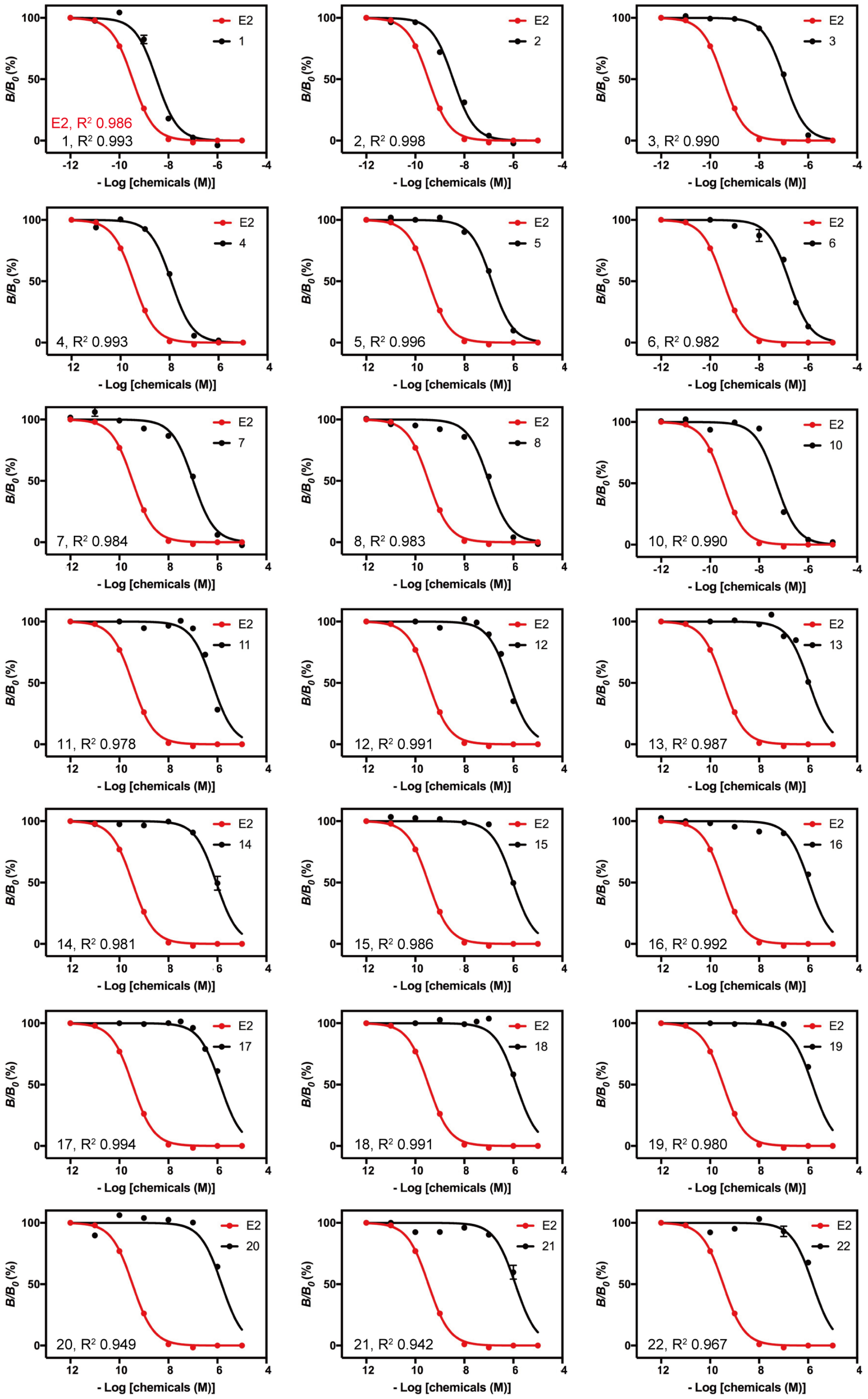


*Supplementary figure 1*

**Supplementary fig. 1. Binding activities of each chemicals by the competitive binding assays.** The representative curves and the $R^2$ values are shown in each graph. $B/B_0$ is the relative inhibitory activity estimated from the calculation of the percentage of displacement by the chemical tested (B) against the specific binding ($B_0$ = 100%) of $[^3H]E2$.

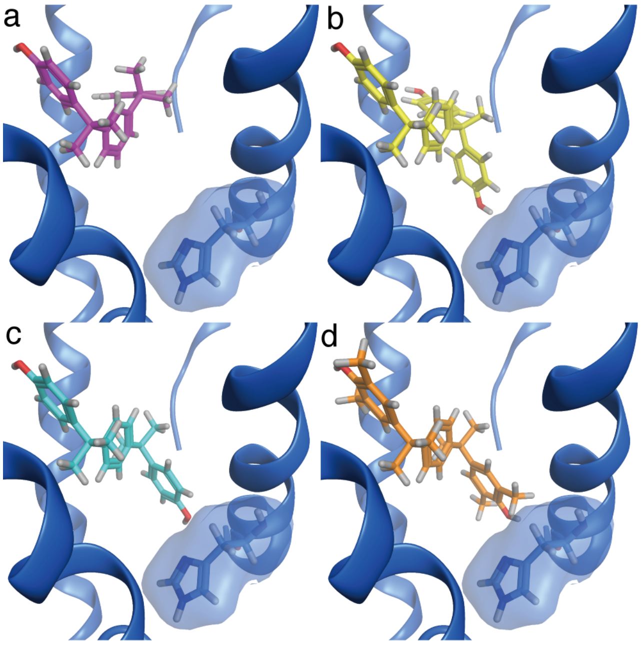


*Supplemenary figure 2*

**Supplementary fig. 2. Molecular superposition of tandem tri-ring bisphenols onto an ERα structure.** Superposition of tandem tri-ring bisphenols and BPC calculated using the *in silico* Molecular Superpose function revealing that tandem tri-ring bisphenols do not clash with the H524 side chain in the inactive ERα conformation (PDB ID: 3UUC). BPM (magenta), α,α,α'-tris(4-hydroxyphenyl)-1-ethyl-4-isopropylbenzene (yellow), BPP (blue), and α,α'-bis(4-hydroxy-3,5-dimethylphenyl)-1,4-diisopropylbenzene (orange) are superposed onto the HO-$C_6H_6$-C-$C_6H_6$ moiety of BPA. The molecular surface of the H524 residue located in close proximity to the N-terminus of Helix 11 is shown in transparent blue.

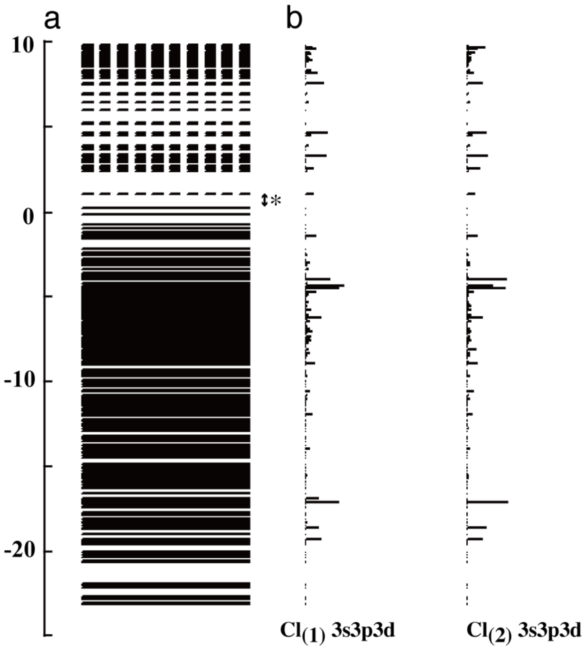


*Supplementary figure 3*

**Supplementary fig. 3. Energy and density diagrams of the HIVE-clipped region in the BPC–ERα complex. a** An example of the calculated energy level diagrams using coordinates clipped 4 Å from a chlorine atom using the HIVE clip method. The energy gap between HOMO and LUMO (HOMO–LIMO gap; indicated as an asterisk) was calculated at 0.9564 eV by *ab initio* experiments in this case. **b** Proportions of filled and unfilled bands comprising of chlorine-3s3p3d orbitals are illustrated as black bars.
